# Whole Body Computed Tomography with Advanced Imaging Techniques: A Research Tool for Measuring Body Composition in Dogs

**DOI:** 10.1155/2013/610654

**Published:** 2013-10-10

**Authors:** Dharma Purushothaman, Barbara A. Vanselow, Shu-Biao Wu, Sarah Butler, Wendy Yvonne Brown

**Affiliations:** ^1^School of Environmental and Rural Science, Department of Animal Science, University of New England, Armidale, NSW 2351, Australia; ^2^NSW Department of Primary Industries, Beef Industry Centre, University of New England, Armidale, NSW 2351, Australia; ^3^North Hill Vet Clinic, Armidale, NSW 2350, Australia

## Abstract

The use of computed tomography (CT) to evaluate obesity in canines is limited. Traditional CT image analysis is cumbersome and uses prediction equations that require manual calculations. In order to overcome this, our study investigated the use of advanced image analysis software programs to determine body composition in dogs with an application to canine obesity research. Beagles and greyhounds were chosen for their differences in morphology and propensity to obesity. Whole body CT scans with regular intervals were performed on six beagles and six greyhounds that were subjected to a 28-day weight-gain protocol. The CT images obtained at days 0 and 28 were analyzed using software programs OsiriX, ImageJ, and AutoCAT. The CT scanning technique was able to differentiate bone, lean, and fat tissue in dogs and proved sensitive enough to detect increases in both lean and fat during weight gain over a short period. A significant difference in lean : fat ratio was observed between the two breeds on both days 0 and 28 (*P* < 0.01). Therefore, CT and advanced image analysis proved useful in the current study for the estimation of body composition in dogs and has the potential to be used in canine obesity research.

## 1. Introduction

Obesity is a common nutritional disorder in dogs with a reported incidence of between 22% and 40% globally [1, 2]. The most commonly used methods to evaluate body composition in canine obesity research are dual-energy X-ray absorptiometry (DXA) and deuterium oxide dilution [3]. When fat estimated by deuterium oxide dilution was validated against fat determined by ether extraction of the carcass using male and female dogs, a coefficient of determination, *r* ^2^ = 0.95, was obtained [4]. When DXA methodology was validated in dogs using chemical analysis of dissected carcasses, it was found to have an overall coefficient of determination, *r* ^2^ = 0.96, for fat mass; however, greater inaccuracies were observed in some individual animals mainly due to skeletal muscle hydration [5]. This was further confirmed in a more recent study in pigs [6] that evaluated the DXA methodology using whole dissection and ashing and concluded that DXA provided inaccurate and misleading results without taking into consideration the hydration and lipid content variability within tissues. A recent study investigated a potentially new method for detecting body composition in dogs: bioimpedance spectroscopy [7]. The method was validated against DXA and found good agreement with the two methods (correlation coefficient *r* = 0.93 for fat) at a population level, but was limited in accuracy when used for individual animal measurements. Quantitative magnetic resonance (QMR) also has been shown to be a useful technique in dogs particularly because the dogs do not require sedation or anaesthesia [8]. 

Computed tomography (CT) works on the principle of acquiring information based on the X-ray radiation being transmitted in many directions through a specific volume of tissue. These transmitted radiations account for the linear attenuation coefficient which are transformed to CT values or Hounsfield units (HU), a quantitative scale for measuring radio-density ranging between −1024 for air, 0 for water, and +1000 for bone, with muscle having a positive HU value, while fat has a negative HU value. From human studies it has been suggested that computed tomography (CT) may be a more accurate method for measuring body composition than DXA [9]. Validation of CT in pigs using dissection and near-infrared spectroscopy showed a coefficient of determination *r* ^2^ = 0.93 [10]. The use of CT to evaluate body composition has been reported for other species: cats [11], minipigs [12], and particularly sheep [13–15], but only one study has utilized CT for measuring body composition in dogs [16]. This study demonstrated that the fat content measured at the third lumbar vertebra (L3) using the attenuation range of −135/−105 HU had the best correlation; correlation coefficient *r* = 0.98, with the body fat content estimated by deuterium oxide dilution method. However, CT slices analyzed were limited to only three levels: 12th thoracic vertebra (T12), the third lumbar vertebra (L3), and the fifth lumbar vertebra (L5), and were only investigated in beagles. The canine study [16] also demonstrated the potential for fat to be overestimated at −190/−30 HU. This further emphasized the need for an improved CT method in dogs. Traditional CT image analysis is cumbersome in a whole body scan because of the large number of CT images involved and the manual calculations required in the prediction equations. The application of advanced image analysis software programs simplifies and automates this process [13]. 

It has been noted that some breeds of dogs [2] are more prone to obesity than others. Therefore, the present study aimed to investigate the use of advanced imaging software techniques with CT to measure body composition in two breeds of dogs. Beagles and greyhounds were chosen because of their differences in morphology and propensity to obesity.

## 2. Materials and Methods

### 2.1. Experimental Animals and Design

Twelve dogs: six beagles and six greyhounds weighing 10.7 ± 0.9 kg (beagles) and 24.7 ± 2.0 kg (greyhounds), were recruited for the study. The veterinarian inspected all the dogs at the commencement of the experiment and only healthy dogs were used. Initial body condition, assessed using a 5-point body condition score, found that all dogs were within ideal range. The dogs were scanned using CT on day 0 and subjected to a weight gain protocol by incorporating saturated fat of coconut oil origin in the diets for 28 days, and whole body scans were repeated. The objective was to determine whether the CT scanning would be sensitive enough to detect fat deposition during weight gain over a short period.

For the duration of the 28-day study, dogs were housed at the University of New England (UNE) dog research facilities at Armidale, NSW, Australia. This study was approved by the University of New England Animal Ethics Committee (Authority no. AEC10/091), and written consent was obtained from the dog owners. All dogs participating in this study were privately owned and were returned to their owners at the end of the study.

### 2.2. Anaesthesia, CT Scanning, and Images

Following an overnight fast, dogs were sedated using medetomidine HCl (Domitor, Pfizer Australia Pty Ltd., West Ryde, NSW, Australia, 1 mg/mL) and butorphanol (Ilium Butorgesic, Troy Laboratories Pty Ltd., Smithfield, NSW, Australia, 10 mg/mL), each administered IV at 0.1 mL per 5 kg bodyweight (BW). Dogs were positioned in sternoabdominal recumbency on a fiberglass cradle lined with foam and gently strapped to prevent movement. A whole body scan with regular intervals was performed using a Picker UltraZ 2000 CT scanner, Philips (Philips Medical Imaging Australia, Sydney, NSW, Australia). The acquisition parameters of the CT scanner were as follows: 120 kV; 100 mA; 480 mm field of view; 5 mm thickness; 10 mm spacing and 1 s scanning time. After scanning, the sedation was reversed using atipamezole HCl (Antisedan, Pfizer Australia Pty Ltd., West Ryde, NSW, Australia, 5 mg/mL, IV) at 0.1 mL per 5 kg BW. Throughout the scanning process, the study veterinarian (SB) monitored the sedation of the dogs. The scanning procedure generated an average of 80 CT images for each beagle and 98 CT images for each greyhound in a single scan, and resulting images were analyzed using software programs—OsiriX, ImageJ, and AutoCAT. 

### 2.3. OsiriX and ImageJ Programs

OsiriX, an open source software [17], was used to edit the digital images obtained from the CT scanner in DICOM format. The use of the OsiriX software program followed the published instructions [18]. Closed polygon region of interest (ROI) was drawn to remove extraneous objects such as the fiberglass cradle from each of the CT images. The area outside the ROI was set to −1024 (air). This new setting deleted the area outside the ROI and allowed ROI to be exported and saved in 16-bit black and white image in DICOM format. The saved images were then processed using ImageJ. 

ImageJ is a public domain image analysis program [19] that can process images in DICOM format [20]. ImageJ was used to convert 16-bit CT images to 8-bit binary images. This modification was a prerequisite for the next image analysis program used: AutoCAT. 

### 2.4. AutoCAT Program and Body Composition

AutoCAT is an automated image analysis program [10] developed using methods similar to the previously developed CATMAN program [15]. AutoCAT program partitions the CT images into fat, lean, and bone based on the HU range for each tissue and measures their area, mean pixel value, and variance. Tissue volumes are then calculated by integrating the area of the respective tissues [21], and tissue densities are calculated using a mathematical function relating HU values to tissue density:

(1) Tissue  density  =  1.0062+(mean  tissue  Hounsfield  unit  value  ×  0.00601) (see   [22]).

**Figure 1:**
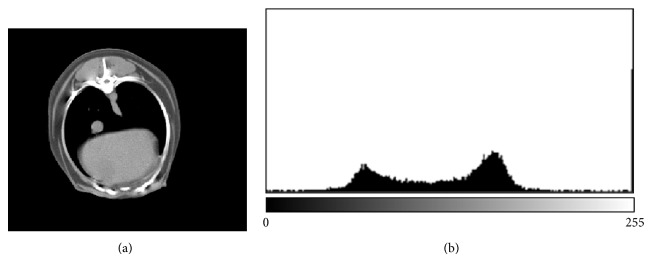
Representative CT image with a histogram to show the ranges in greyscale units 20–130 (fat), 131–220 (lean), and 221–255 (bone).

AutoCAT then calculates tissue weight from the volume and density measurements. An additional function called CALC within the AutoCAT program calculated the total weight of lean, fat, and bone for each animal [10] using new ranges that were manually set to 20–130 (fat), 131–220 (lean), and 221–255 (bone) in greyscale units. These ranges were chosen for the canine species specifically in our study based on the histogram analysis in ImageJ program (see Figure 1). The equivalent values of the greyscale units in HU units were −214 to +7 for fat, 8 to 187 for lean, and 188 to 3072 for bone. These HU ranges were determined by the following formula:

(2) $$\begin{array}{c} \text{HU}=2*\text{GU}-254\;\left(\text{see}\;\left[23\right]\right), \end{array}$$

wherein GU is the greyscale value out of AutoCAT. The factor of 2 was used in the formula as 2 GU values were being combined into one HU value. The offset of 254 was an intercept adjustment to set water in the middle of a 256 GU range. 

For clarification, total bodyweights obtained in this study from AutoCAT have been designated as CT-derived BW. The same researcher performed the various steps of CT image analysis to avoid biased analysis. 

### 2.5. Bodyweight Measurement

Prior to CT scanning, the dogs were weighed on an electronic weigh scale, Provet Nuweigh Scales CHR-592 (Provet VMS Pty Ltd., Cameron Park, NSW, Australia) which was calibrated against a known weight before the initial use. The scales were set to “zero” before each weighing session, and dogs were weighed thrice to confirm the weight obtained. Throughout this study, the BW obtained from the electronic weigh scale has been designated as measured BW.

### 2.6. Statistical Analysis

Bland-Altman (BA) test of agreement was used to analyze the relationship between CT-derived BW and measured BW. To compare the differences in lean : fat ratios between the breeds, Mann-Whitney *U* test was used as the data was nonparametric in nature. MedCalc (MedCalc, Mariakerke, Belgium, Version 12.7.1.0) was used for statistical analysis, and *P* < 0.05 was considered statistically significant.

## 3. Results

### 3.1. Differences in Body Composition in Beagles and Greyhounds

For both beagles and greyhounds there was a nonsignificant increase in both lean and fat over the 28 days. Body composition data are presented in Table 1. A significant difference in the lean : fat ratio was seen between the two breeds on both days 0 and 28 (*P* < 0.01, Mann-Whitney *U* test). 

### 3.2. CT-Derived BW versus Measured BW

Total BW were determined using the two methods CT-derived BW and measured BW described previously were compared. A Bland-Altman test of agreement (Table 2) shows that the two methods are interchangeable with respect to the BW as all the data points are within the mean ± 1.96 SD and that the BA ranges for the two measurements are not wide.

## 4. Discussion

The CT imaging and data analysis used in this study were able to differentiate bone, lean, and fat tissue in dogs. The CT scanning technique was sensitive enough to detect increases in both lean and fat during weight gain over a short period. In addition, the lean : fat ratio decreased in all dogs consistently with fat deposition. Importantly, the bone weights estimated by CT were identical on days 0 and 28 for each animal, supporting the reliability and repeatability of this technique.

The results obtained demonstrated a significant relationship between the CT-derived body weight and measured body weight, with the capacity to differentiate body composition such as lean : fat ratio in the two breeds of dogs. The significant relationship between the two methods of bodyweight determination is similar to a study reported in sheep using the same research method wherein coefficient of determination, *r* ^2^ = 0.96, was observed when liveweight was correlated with CT estimates of liveweight [13]. In the present study, a consistent underestimation of CT-derived body weight was observed. This is partially similar to the findings of the sheep study wherein the author accounted for the underestimation to be due to the head and feet not being included in the CT analysis and the wool also not being accounted for as it does not absorb X-rays [13]. An underestimation was also reported in a pig study wherein the AutoCAT program was used to estimate the final weights of the fat, bone, and lean tissue [10]. In the pig study, CT-derived bone weight was significantly underestimated, lean tissue was significantly overestimated, when compared to weights measured by a combination of dissection and NIR, and fat was underestimated, though not significantly. The author accounted for the differences and inaccuracies in bone, and lean tissue weights due to the fundamental differences between the two methods that were compared, CT and combination of dissection and NIR. It is not clear why a consistent underestimation was observed in the present study in dogs. However, it is possible that the observed underestimation could have been due to the noncontinuous nature of the CT slices. In addition, it is also not clear if the algorithm of the AutoCAT program previously tested in sheep and pigs requires a slight modification specifically for the canine species. Future work could involve a whole body scan with no gap between the slices, but this would increase the exposure time of the dogs to radiation and increase the number of CT slices for analyses.

The present study estimated body composition in dogs (total body fat, lean tissue, and bone), which was achieved using novel image analysis software program—AutoCAT together with software programs (OsiriX and ImageJ) to enhance the image analysis capabilities. When individual dogs were scanned on day 28, no change was seen in the estimated bone weight compared to day 0. This further emphasized the accuracy of the AutoCAT program. The use of AutoCAT program has been reported previously in sheep [14, 24, 25] and highlighted the diverse potential of the AutoCAT program for predicting body composition without the need of a validated prediction equation. This is the first study to report whole body CT scanning for measuring body composition in dogs. The challenge in developing an application of CT for body fat estimation is that the attenuation range set for fat can underestimate or overestimate the fat content. In the previously published CT study using beagles, the attenuation range for fat was proposed as −135/−105 [16]. In the present study wherein two different breeds were used, HU for fat was set as −214 to +7. This larger attenuation range for fat was chosen with the use of ImageJ software program to assist in detecting breed differences in fat composition. The CT techniques used in the present study demonstrated a significant breed difference in the lean : fat ratio. 

Although there are advantages in applying CT to estimate body composition in dogs, the costs involved and the need to sedate the dogs limit the use of this methodology in a clinical setting. It is also recognized that the design of the present study could be strengthened with the inclusion of a comparison and validation with other existing methods, such as DXA and deuterium oxide dilution. Another aspect of body fat estimation, not explored in the present study, is the estimation of visceral/subcutaneous ratio (V/S). AutoCAT software program has demonstrated the potential to estimate the total weight in kg of subcutaneous fat, as well as intramuscular fat in sheep [14]. Hence, future studies in canines using the present CT methodology should explore V/S ratio to determine visceral obesity, as it has been linked to metabolic diseases [26]. 

## 5. Conclusion

The findings of this study indicate that CT combined with advanced image analysis software is a promising candidate for an alternative noninvasive method for assessing body composition in dogs.

## Figures and Tables

**Table 1:** Body composition of individual dogs on day 0 and day 28.

Dog no.	Day	Bone (kg)	Lean (kg)	Fat (kg)	Lean/fat
Beagle 1	0 28	1.7 1.7	6.0 6.7	3.9 4.8	1.5 1.4
Beagle 2	0 28	1.8 1.8	6.4 7.4	2.5 3.4	2.6 2.2
Beagle 3	0 28	1.6 1.6	5.6 6.2	2.7 3.3	2.1 1.9
Beagle 4	0 28	1.7 1.7	7.3 7.9	2.8 3.1	2.6 2.5
Beagle 5	0 28	1.4 1.4	5.8 6.1	3.2 3.9	1.8 1.6
Beagle 6	0 28	1.5 1.5	6.1 6.6	2.3 2.9	2.7 2.3

Greyhound 1	0 28	3.5 3.5	19.7 20.3	3.5 4.0	5.6 5.1
Greyhound 2	0 28	3.2 3.2	17.4 18.3	1.7 2.6	10.2 7.0
Greyhound 3	0 28	3.4 3.4	20.2 21.0	2.6 3.9	7.8 5.4
Greyhound 4	0 28	3.4 3.4	19.5 20.3	3.5 4.0	5.6 5.1
Greyhound 5	0 28	3.0 3.0	17.1 17.7	2.6 3.2	6.6 5.5
Greyhound 6	0 28	3.3 3.3	19.0 20.0	2.0 3.5	9.5 5.7

Beagles (mean ± SD)	0 28	1.6 ± 0.1 1.6 ± 0.1	6.2 ± 0.6 6.8 ± 0.7	2.9 ± 0.6 3.6 ± 0.7	2.2 ± 0.5 2.0 ± 0.4
Greyhounds (mean ± SD)	0 28	3.3 ± 0.2 3.3 ± 0.2	18.8 ± 1.3 19.6 ± 1.3	2.7 ± 0.7 3.5 ± 0.6	7.5 ± 2.0 5.6 ± 0.7

**Table 2:** Comparison of measured and CT-derived bodyweights (BW) using Bland-Altman (BA) test of agreement.

	Measured BW ± SD (kg)	CT-derived BW ± SD (kg)	Mean difference ± SD (kg)	BA limits (kg)	BA range (kg)	*P *
Beagles day 0	11.3 ± 1.0	10.7 ± 0.8	−0.63 ± 0.14	−0.90 to −0.37	−0.54	<0.0001
Beagles day 28	12.6 ± 1.1	12.0 ± 0.9	−0.58 ± 0.19	−0.96 to −0.20	−0.76	0.0035
Greyhounds day 0	25.8 ± 1.8	24.8 ± 2.0	−0.98 ± 0.24	−1.45 to −0.51	−0.94	0.0001
Greyhounds day 28	27.7 ± 1.9	26.4 ± 1.9	−1.30 ± 0.26	−1.81 to −0.79	−1.02	<0.0001

## References

[1] German A. J. (2006). The growing problem of obesity in dogs and cats. *Journal of Nutrition*.

[2] Edney A. T., Smith P. M. (1986). Study of obesity in dogs visiting veterinary practices in the United Kingdom. *The Veterinary Record*.

[3] Mawby D. I., Bartges J. W., d'Avignon A., Laflamme D. P., Moyers T. D., Cottrell T. (2004). Comparison of various methods for estimating body fat in dogs. *Journal of the American Animal Hospital Association*.

[4] Burkholder W. J., Thatcher C. D. (1998). Validation of predictive equations for use of deuterium oxide dilution to determine body composition of dogs. *American Journal of Veterinary Research*.

[5] Speakman J. R., Booles D., Butterwick R. (2001). Validation of dual energy X-ray absorptiometry (DXA) by comparison with chemical analysis of dogs and cats. *International Journal of Obesity*.

[6] Clarys J. P., Scafoglieri A., Provyn S., Louis O., Wallace J. A., De Mey J. (2010). A macro-quality evaluation of DXA variables using whole dissection, ashing, and computer tomography in pigs. *Obesity*.

[7] Ward L. C., Rae L., Vankan D., Flickinger E., Rand J. Prediction of body composition in dogs by bioimpedance spectroscopy.

[8] Zanghi B. M., Cupp C. J., Pan Y. (2013). Noninvasive measurements of body composition and body water via quantitative magnetic resonance, deuterium water, and dual-energy x-ray absorptiometry in awake and sedated dogs. *American Journal of Veterinary Research*.

[9] Lane J. T., Mack-Shipman L. R., Anderson J. C. (2005). Comparison of CT and dual-energy DEXA using a modified trunk compartment in the measurement of abdominal fat. *Endocrine*.

[10] Jopson N. B., Kolstad K., Sehested E., Vangen O. (1995). Computed tomography as an accurate and cost effective alternative to carcass dissection. *Proceeding of the Australian Association Animal Breeding and Genetics*.

[11] Buelund L. E., Nielsen D. H., Mcevoy F. J., Svalastoga E. L., Bjornvad C. R. (2011). Measurement of body composition in cats using computed tomography and dual energy x-ray absorptiometry. *Veterinary Radiology and Ultrasound*.

[12] Chang J., Jung J., Lee H., Chang D., Yoon J., Choi M. (2011). Computed tomographic evaluation of abdominal fat in minipigs. *Journal of Veterinary Science*.

[13] Haynes F. E. M., Greenwood P. L., Siddell J. P., McDonagh M. B., Oddy V. H. (2010). Computer tomography software program “Osirix” aids prediction of sheep body composition. *Proceedings of the Australian Society of Animal Production*.

[14] Kvame T., Vangen O. (2007). Selection for lean weight based on ultrasound and CT in a meat line of sheep. *Livestock Science*.

[15] Kinghorn B., Thompson J. (1992). CATMAN—a program to measure CAT-Scans for predictions of body components in live animals. *Proceeding Australian Association Animal Breeding and Genetics*.

[16] Ishioka K., Okumura M., Sagawa M., Nakadomo F., Kimura K., Saito M. (2005). Computed tomographic assessment of body fat in beagles. *Veterinary Radiology and Ultrasound*.

[17] Rosset A., Spadola L., Ratib O. (2004). OsiriX: an open-source software for navigating in multidimensional DICOM images. *Journal of Digital Imaging*.

[18] Ratib O., Rosset A., Heuberger J. (2002). *OsiriX: The Pocket Guide*.

[19] Abràmoff M. D., Magalhães P. J., Ram S. J. (2004). Image processing with imageJ. *Biophotonics International*.

[20] Escott E. J., Rubinstein D. (2003). Free DICOM image viewing and processing software for your desktop computer: what's available and what it can do for you. *Radiographics*.

[21] Gundersen H. J. G., Bendtsen T. F., Korbo L. (1988). Some new, simple and efficient stereological methods and their use in pathological research and diagnosis. *Acta Pathologica, Microbiologica, et Immunologica Scandinavica*.

[22] Fullerton G. D., Fullerton G. D., Zagzebski J. A. (1980). Tissue imaging and characterisation. *Medical Physics of CT and Ultrasound. Medical Physics Monograph*.

[23] Jopson N. B. (1993). *Physiological adaptations in two seasonal cervids [dissertation]*.

[24] Kvame T., Vangen O. (2006). In-vivo composition of carcass regions in lambs of two genetic lines, and selection of CT positions for estimation of each region. *Small Ruminant Research*.

[25] Haynes F. E. M., Greenwood P. L., McDonagh M. B., Oddy V. H. (2012). Myostatin allelic status interacts with level of nutrition to affect growth, composition, and myofiber characteristics of lambs. *Journal of Animal Science*.

[26] Bergman R. N., Kim S. P., Catalano K. J. (2006). Why visceral fat is bad: mechanisms of the metabolic syndrome. *Obesity*.

